# A Neoantigen-Based Peptide Vaccine for Patients With Advanced Pancreatic Cancer Refractory to Standard Treatment

**DOI:** 10.3389/fimmu.2021.691605

**Published:** 2021-08-13

**Authors:** Zheling Chen, Shanshan Zhang, Ning Han, Jiahong Jiang, Yunyun Xu, Dongying Ma, Lantian Lu, Xiaojie Guo, Min Qiu, Qinxue Huang, Huimin Wang, Fan Mo, Shuqing Chen, Liu Yang

**Affiliations:** ^1^Cancer Center, Department of Medical Oncology, Zhejiang Provincial People’s Hospital, People’s Hospital of Hangzhou Medical College, Hangzhou, China; ^2^Hangzhou Neoantigen Therapeutics Co., Ltd., Hangzhou, China; ^3^Zhejiang California International Nanosystems Institute, Zhejiang University, Hangzhou, China; ^4^Department of Gastrointestinal and Pancreatic Surgery, Zhejiang Provincial People’s Hospital, People’s Hospital of Hangzhou Medical College, Hangzhou, China; ^5^College of Pharmaceutical Sciences, Zhejiang University, Hangzhou, China; ^6^Vancouver Prostate Centre, University of British Columbia, Vancouver, BC, Canada; ^7^Hangzhou AI-Force Therapeutics Co., Ltd., Hangzhou, China

**Keywords:** neoantigen, pancreatic cancer, vaccine, peptide, immunotherapy

## Abstract

**Background:**

Neoantigens are critical targets to elicit robust antitumor T-cell responses. Personalized cancer vaccines developed based on neoantigens have shown promising results by prolonging cancer patients’ overall survival (OS) for several cancer types. However, the safety and efficacy of these vaccine modalities remains unclear in pancreatic cancer patients.

**Methods:**

This retrospective study enrolled 7 advanced pancreatic cancer patients. Up to 20 neoantigen peptides per patient identified by our in-house pipeline iNeo-Suite were selected, manufactured and administered to these patients with low tumor mutation burden (TMB) (less than 10 mutations/Mb). Each patient received multiple doses of vaccine depending on the progression of the disease. Peripheral blood samples of each patient were collected pre- and post-vaccination for the analysis of the immunogenicity of iNeo-Vac-P01 through ELISpot assay and flow cytometry.

**Results:**

No severe vaccine-related adverse effects were witnessed in patients enrolled in this study. The mean OS, OS associated with vaccine treatment and progression free survival (PFS) were reported to be 24.1, 8.3 and 3.1 months, respectively. Higher peripheral IFN-γ titer and CD4^+^ or CD8^+^ effector memory T cells count post vaccination were found in patients with relatively long overall survival. Remarkably, for patient P01 who had a 21-month OS associated with vaccine treatment, the abundance of antigen-specific TCR clone drastically increased from 0% to nearly 100%, indicating the potential of iNeo-Vac-P01 in inducing the activation of a specific subset of T cells to kill cancer cells.

**Conclusions:**

Neoantigen identification and selection were successfully applied to advanced pancreatic cancer patients with low TMB. As one of the earliest studies that addressed an issue in treating pancreatic cancer with personalized vaccines, it has been demonstrated that iNeo-Vac-P01, a personalized neoantigen-based peptide vaccine, could improve the currently limited clinical efficacy of pancreatic cancer.

**Clinical Trial Registration:**

ClinicalTrials.gov, identifier (NCT03645148).

Registered August 24, 2018 - Retrospectively registered

## Introduction

Pancreatic cancer is one of the top-leading causes of cancer-related death in the world, with a 5-year survival rate of only 9.3% (1). Most of the pancreatic cancer patients are diagnosed at an advanced stage (2). The poor prognosis of pancreatic cancer mainly results from the lack of early detection strategies such as a screening test, as no screening test has yet been shown to lower the risk of dying from pancreatic cancer. Even for those initially diagnosed at an early stage and subsequently received standard treatments such as surgical resection in combination with systemic radiotherapy or chemotherapy, their 5-year OS rate is still below 25% (3). According to the 2018 International Cancer Research Institute (IARC) GLOBOCAN statistics, there were 458,918 new cases and 432,242 deaths of pancreatic cancer, accounting for 2.5% of total new cancer cases and 4.5% of total deaths caused by all cancer types respectively in 2018 (4). Greater efforts should be addressed to the development of more promising therapies for pancreatic cancer.

With the development of chimeric antigen receptor T cell (CAR-T) immunotherapy and immune checkpoint inhibitors (ICIs) such as anti-PD-1/PD-L1 antibodies, cancer immunotherapy has shown attractive potential in the treatment of various solid tumors (5, 6). However, for pancreatic cancer, ICIs alone or combined with chemotherapy have not achieved evident positive outcomes in clinical studies (7, 8). Therapeutic neoantigen cancer vaccines belong to another important category of cancer immunotherapy. Several clinical studies have been launched recently to study their safety, tolerability and efficacy amongst patients diagnosed with different cancer types (9–11). Mostly generated from non-synonymous mutations specific in cancer cells, neoantigens are usually exempted from central tolerance. Personalized peptide neoantigen vaccines designed to train a patient’s immune system can target and kill tumor cells specifically through following steps: deliver neoantigens to antigen-presenting cells (APCs); present tumor-specific neoantigens to T cells and activate cytotoxic T cells to recognize and eliminate tumor cells (12). Activated tumor-specific cytotoxic T-lymphocytes could infiltrate into tumors, turning “cold” tumors into “hot” ones, thereby eliciting a stronger antitumor immune response. Neoantigens of high immunogenicity and abundant CD8^+^ T-cell infiltrates have been detected in long-term survivors of pancreatic ductal adenocarcinoma, suggesting that neoantigen-based cancer immunotherapies could benefit the survival of pancreatic cancer patients (13).

Recently, studies by Wu and Sahin et al. have demonstrated that peptide- or RNA-based neoantigen vaccines not only induce significant regression of advanced melanoma, but also provide long-term protection against tumor relapse and metastasis (14, 15). Sustained T cell response and increase in the number of tumor-infiltrating T cells were also reported in newly diagnosed glioblastoma patients after personalized peptide neoantigen vaccination (16). Studies focusing on colon and esophageal cancer also confirmed the effectiveness of neoantigen vaccination (17, 18). Moreover, combination treatment of personalized peptide neoantigen vaccines with ICIs has demonstrated good feasibility, safety and immunogenicity in patients with advanced melanoma, non-small cell lung cancer as well as bladder cancer in a phase Ib study (11). Although tumor-associated antigen (TAA)-based vaccines have been extensively investigated for their efficacy for pancreatic cancer (19–21), the anticancer effects of personalized neoantigen vaccines remain unclear.

Herein, we retrospectively assessed the anticancer effects of a personalized peptide neoantigen cancer vaccine, iNeo-Vac-P01, in patients with advanced pancreatic cancer from a clinical study (trial number: NCT03645148). Comprehensive analysis of these patients’ immune response after vaccination was done to investigate its safety, tolerability and anticancer efficacy.

## Methods

### Patients

Eligible patients with advanced pancreatic cancer confirmed histologically or cytologically were aged at least 18 years. Only patients who developed chemotherapy intolerance or disease progression after second-line treatments, with at least one measurable lesion in accordance with investigator-assessed Response Evaluation Criteria in Solid Tumors (RECIST; version 1.1), an Eastern Cooperative Oncology Group (ECOG) performance status of 0 or 1, as well as physiologically functional healthy organs such as heart, liver and kidney were considered. All patients selected for this study provided sufficient tumor tissue and blood samples for whole exome sequencing (WES) and RNA sequencing (when fresh tumor tissue is available).

Patients who had other malignant tumors except for cured basal cell carcinoma, thyroid carcinoma or cervical dysplasia, who lacked identified neoantigens by sequencing, who had received bone marrow or stem cell transplant or were allergic to polypeptides or other immunotherapies were excluded from this study.

### Study Design and Treatment

We retrospectively investigated the clinical response of advanced pancreatic cancer patients upon receiving a personalized neoantigen peptide vaccine from a single-arm, open-label and investigator-initiated clinical study at Zhejiang Provincial People’s Hospital in China (NCT03645148). The primary endpoints of this clinical study were safety and feasibility, which were assessed based on the occurrence of adverse events (AEs) and whether the identification of neoantigens by our in-house pipeline iNeo-Suite and the subsequent peptide synthesis could be accomplished for clinical use. Whereas the secondary endpoint was efficacy which was evaluated through progression-free survival (PFS), overall survival (OS) and neoantigen-specific immune responses.

All patients received neoantigen vaccine iNeo-Vac-P01 comprising 5~20 peptides of varying length of 15 to 35 amino acids multiple times depending on the progression of the disease. Based on their HLA typing, affinity and allele frequency, these peptides were grouped into 2~4 pools and administered subcutaneously (s.c.) at the dose of 100 μg per peptide to patients at their upper arms and paraumbilical area. Each patient was primed with iNeo-Vac-P01 on day 1, 4, 8, 15 and 22, and boosted with the same vaccine formulation on day 78 and 162. Additional booster shots were scheduled to some patients to maximize the clinical benefits in accordance with the clinical research protocol. Thirty minutes prior to each immunization, 40 μg granulocyte-macrophage colony-stimulating factor (GM-CSF) was administered subcutaneously to patients around the injection site as an adjuvant (14, 16, 22–24). Poly-IC was tested in our previous study, however, due to the observation of AEs in patients, GM-CSF was chosen as an adjuvant for this study. Application of concomitant medical therapy such as ICIs during neoantigen vaccination was determined by clinicians to improve each patient’s clinical response in accordance with the clinical research protocol. The treatment regimen for each patient was summarized in **Supplementary Table 1**.

Clinical assessment, monitoring and follow-up consisted of physical examination such as ECOG performance, vital sign, blood test and urinalysis for safety evaluation, imaging examination at baseline and post-vaccination for efficacy assessment, as well as immune response testing such as IFN-γ Enzyme-Linked Immunospot (ELISpot) assay and flow cytometry (T cell subsets and cytokines) at pre- and post-vaccination stages.

A retrospective assessment of tumors was conducted by investigators at each time point (baseline and approximately every 8 weeks thereafter) according to RECIST v1.1 criterion. The clinical response of each patient was evaluated not only throughout the vaccination, but also at regular intervals of 3 months post-vaccination until the development of cumulative toxic effects, disease progression or discontinuation of treatment. The occurrence of adverse events (AEs) were recorded, with the severity graded in accordance with National Cancer Institute Common Terminology Criteria for Adverse Events (version 4.0) throughout the treatment.

The study protocol was approved by the Institutional Review Board and Independent Ethics Committee and implemented in accordance with the Declaration of Helsinki and the International Conference on Harmonization Guidelines for Good Clinical Practice. All patients had signed informed consent forms before immunization.

### Generation of Personalized Peptide Neoantigen Vaccines

To identify mutation-derived neoantigens, whole exome sequencing was conducted on samples obtained from patients by surgery, biopsy or intravenous blood sampling using Hiseq 4000 NGS platforms (Illumina) with coverage depths of 500x for tumor cells and 100x for blood cells (Novogene Biotech Co., Ltd., Beijing, China) (25–29). In addition, formalin-fixed paraffin-embedded (FFPE) samples were used for WES when fresh tumor samples were unavailable.

Bioinformatic analysis was performed by our in-house pipeline iNeo-Suite consisting of multiple modules including sequencing read filtering, genome alignment, mutation calling, HLA typing, MHC affinity prediction, gene expression profiling, vaccine peptide sequence design and mutation-centered prioritization based on therapeutic potency (Supplementary Methods).

Customized clinical-grade long peptides were manufactured through chemical synthesis at GMP-like standard (bacteria-free, > 95.0% purity with endotoxin less than 10 EU/mg) to generate iNeo-Vac-P01. The water solubility of synthesized peptides was tested, and water insoluble peptides were excluded from the final formulation.

### IFN-γ Enzyme-Linked Immunospot Assay

To confirm the immunogenicity of iNeo-Vac-P01, ELISpot assays were performed for each patient at multiple time points pre- and post-vaccination. Peripheral blood (10-30 mL) was obtained from each patient for the isolation of peripheral blood mononuclear cells (PBMCs). PBMCs were then co-incubated (2×10^5^ cells per well) with peptides for 16-24 hours using human IFN-γ pre-coated ELISpot kit following the standard protocol. Spots in ELISpot plates were counted using an automatic plate reader with proper parameters (Supplementary Methods).

### T Cell Receptor Sequencing

To monitor the change of T cell population for each patient, T cell receptor (TCR) β chains were sequenced before and after vaccination. RNA extraction of PBMCs was performed using RNeasy Plus Mini Kit (Qiagen). Samples were analyzed by High-throughput sequencing of TCR using ImmuHub TCR profiling system at a deep level (ImmuQuad Biotech). Briefly, a 5’ RACE unbiased amplification protocol was used. Unique molecular identifiers (UMIs) introduced to the course of cDNA synthesis were used to control bottlenecks and eliminate the errors of PCR and sequencing. Sequencing was performed on an Illumina HiSeq system with PE150 mode (Illumina). One common adaptor with UMI was added to the 5’ of cDNA during the synthesis of first-strand cDNA. One reverse primer corresponding to the constant (C) regions of each TCRα and β was designed to facilitate PCR amplification of cDNA sequences in a less biased manner. The UMIs attached to each raw sequence reads were applied for sequencing error correction and PCR duplication removal. V, D, J and C segments were mapped with IMGT. CDR3 regions were extracted, and clonotype assembled for all clones. The special nucleotide/amino acid sequences of CDR3 region of TCRβ subunit were determined. Those with out-of-frame or stop codon sequences were removed from the identified TCRβ repertoire. The total number of TCRβ clones sharing the same nucleotide sequence of CDR3 region was defined as the amount of each TCRβ clonotype.

### Cytometric Analysis of T-Lymphocyte and Cytometric Bead Array Analysis of Cytokines

To quantify the activation of T cells after vaccination, peripheral T cells extracted from each patient were labeled with several different antibodies (CD279, CD197, CD4, CD8, CD45RA, CD38, CD45, CD3, HLA-DR and CD152) for flow cytometry analysis. To examine the cytokines secreted from activated T cells after vaccination, cytokine titers in peripheral blood were measured by CBA following manufacturer’s protocol (Supplementary Methods).

### Statistical Analysis

Data from the patients who received at least one dose of iNeo-Vac-P01 was analyzed for safety and clinical efficacy assessment. Descriptive statistics was applied to determine the characteristics of baseline and assess the safety of iNeo-Vac-P01. The target lesions of each patient were measured before the treatment and then every two months during the treatment to monitor the changes in lesion sizes. All tumors were sized by MRI and CT. Disease control rate (DCR) was defined as the proportion of patients who had complete response (CR), partial response (PR) and stable disease (SD) for best clinical response. Standard RECISTv1.1 guideline was followed for the analysis of all clinical data. The survival curves were plotted by GraphPad Prism 5 (v5.01).

## Results

### Patients and Demographics

A total of 7 eligible advanced pancreatic cancer patients, enrolled between January 1, 2018 to March 31, 2020, were included in this retrospective study. All patients had previously received surgery or standard chemotherapy, and experienced cancer relapse or metastasis. Patients baseline characteristics were summarized in **Table 1**. Six (85.71%) patients had adenocarcinoma and one (14.29%) had squamous cell carcinoma. Four (57.14%) patients had liver metastases and three (42.86%) had peritoneum metastases. In addition, four (57.14%) patients had higher CA19-9 levels at baseline compared to the other three (42.86%) with normal levels.

**Table 1:** Baseline characteristics of each patient.

Characteristics	Patient (N=7)	
	n	%
**Sex**		
Male	5	71.43
Female	2	28.57
**Age**		
<60	3	42.86
>=60	4	57.14
**Tumor location**		
Pancreatic head	2	28.57
Pancreatic body and tail	5	71.43
**Histopathology**		
Adenocarcinoma	6	85.71
Others	1	14.29
**Metastatic sites**		
Liver	4	57.14
Peritoneum	3	42.86
**ECOG score**		
0	3	42.86
1	4	57.14
**CA19-9 level(first visit)**		
<37.0	3	42.86
>=37.0	4	57.14

ECOG, Eastern cooperative oncology group; CA19-9, Carbohydrate antigen 19-9.

### Feasibility of Preparation and Application of Neoantigen in Patients With Advanced Pancreatic Cancer

WES results of both tumor tissues and peripheral blood samples were shown in **Supplementary Table 2**. Neoantigens were predicted and prioritized using our in-house pipeline iNeo-Suite, in consideration of allelic frequency of mutation, affinity between mutated peptide and HLA class I and II, as well as feasibility of peptide synthesis (**Supplementary Tables 3**, **4**). Clinical-grade long peptides (15-35 amino acids) incorporating multiple neo-epitopes of both HLA class I and II were synthesized (**Supplementary Table 3**). Depending on the quantities of tumor samples as well as the sequences of long peptides, the turnaround time of the whole process varied from 1.5 to 3 months.

Based on the analysis of 1061 pancreatic cancer samples from cBioPortal for Cancer Genomics, KRAS, TP53, SMAD4 and CDKN2A are the most common mutations in tumor-related genes, and all detected in over 10% patient samples. In this study, the numbers of the patients with TP53, KRAS, SMAD4 and CDKN2A mutations were seven (100%), five (71.43%), three (42.86%) and one (14.29%). Importantly, among patients with KRAS mutations, there were two with G12V mutation, two with G12D and one with Q61H. Despite of the lower tumor mutation burden (TMB) in pancreatic cancers, sufficient neoantigens were identified, followed by successful manufacture of corresponding personalized long peptides for each patient in this study. Different from other clinical studies published prior to this study (14, 16), no organic solvent such as DMSO was applied to enhance the water solubility of personalized long peptides due to the disapproval of its use in clinics. Among the total 94 long peptides that were designed and synthesized successfully, 70 peptides with good water solubility were further selected for vaccination (**Supplementary Table 5**). The median number of peptides administered to each patient was 12. Most patients (5 out of 7) received vaccines consisted of more than 10 peptides (**Supplementary Tables 3**, **5**), which contained a median of 9 class I neo-epitopes and 20 class II neo-epitopes per peptide.

### Treatment and Follow-Up of Patients

**Figure 1:**
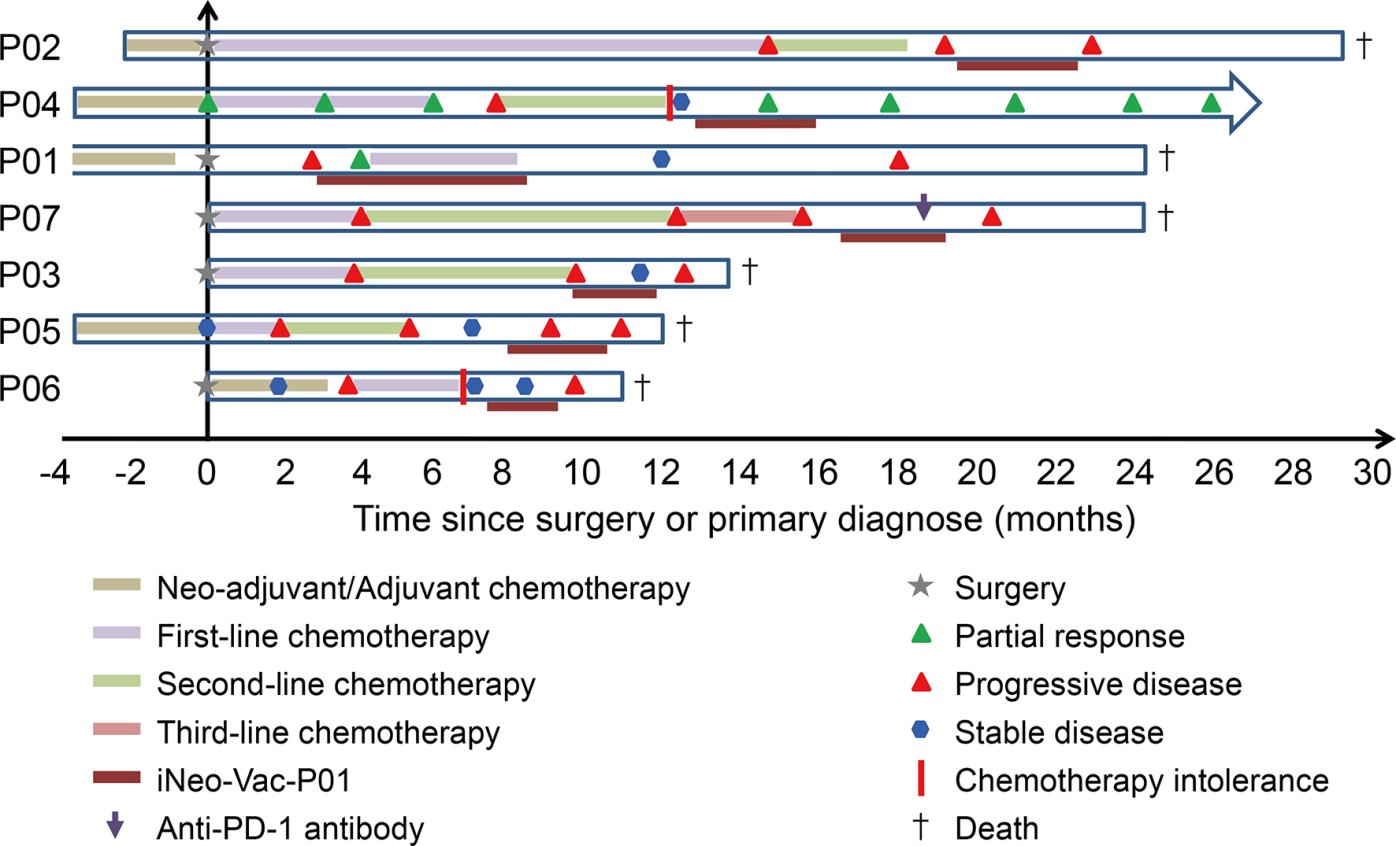
Clinical treatment process for each patient from surgery or primary visit until the end of follow-up.

Patients were scheduled to receive iNeo-Vac-P01 together with GM-CSF as adjuvant (**Figure 1**). The median follow-up duration for all patients was 9.7 months, ranging from 2 months to 21 months before the deadline March 31, 2020. All patients completed the prime phase of immunization (**Figure 1**). The average duration of treatment was 2.57 months, ranging from 1 month to 5 months. Patients P02, P03, P06 and P07 had stable disease during vaccination. Patients P01 and P04 showed partial response during vaccination and had good disease control for a period after vaccination. Patients P05 had progressive disease (PD) during the boost phase of vaccination.

### Safety and Side Effects

During the vaccine treatment, none of the patients had grade 3-4 adverse events associated with iNeo-Vac-P01 defined by NCI CTCAE 4.03. One of the patients (P06) experienced a mild rash after vaccine injection but recovered within one week. To be noted, all seven patients had experienced different degrees of adverse reactions due to chemotherapy before scheduled for vaccination. The most common serious adverse events of chemotherapy among these patients were hematological toxic events including neutropenia (7/7) and anemia (5/7). Other chemotherapy-related adverse reactions including gastrointestinal reactions, rashes and fever were summarized in **Table 2**.

**Table 2:** Comparison of adverse reactions between peptide therapy and chemotherapy in treated patients.

Adverse Effects	Any grade		Grades 3 to 4	
	Chemotherapy	Neoantigen vaccine	Chemotherapy	Neoantigen vaccine
	N (%)	N (%)	N (%)	N (%)
Gastrointestinal reaction	7 (100.0)	0 (0.0)	2 (28.6)	0 (0.0)
Thrombocytopenia	5 (71.4)	0 (0.0)	2 (28.6)	0 (0.0)
Rash	2 (28.6)	1 (14.3)	0 (0.0)	0 (0.0)
Fever	1 (14.3)	0 (0.0)	0 (0.0)	0 (0.0)
Neutropenia	7 (100.0)	0 (0.0)	4 (57.1)	0 (0.0)
Peripheral nerve abnormalities	3 (42.9)	0 (0.0)	0 (0.0)	0 (0.0)
Anemia	5 (71.4)	0 (0.0)	1 (14.3)	0 (0.0)
Elevated transaminase	3 (42.9)	0 (0.0)	0 (0.0)	0 (0.0)
Fatigue	7 (100.0)	0 (0.0)	1 (14.3)	0 (0.0)

### Clinical Response

**Figure 2:**
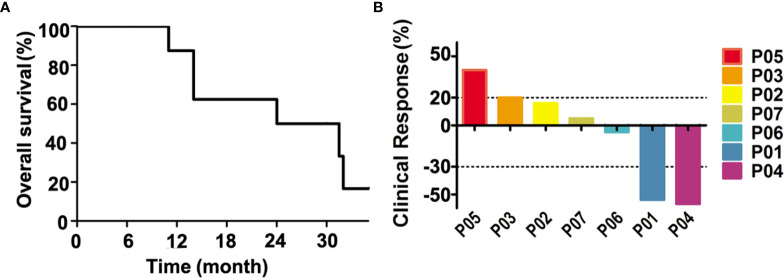
The clinical response and prognosis of treated patients. **(A)** The overall survival curve of each patient. **(B)** The percentage changes of tumor lesion in size from baseline. The changes in lesions between a positive value of 25% and a negative value of 50% are considered stable disease.

RECIST 1.1 criteria were used to assess target lesions in all patients. The OS of all patients was summarized in **Figure 2A**. The mean OS of the 7 patients was 24.1 months (11 to 31.4 months), and the mean PFS was 3.1 months (**Table 3**). Calculated from each patient’s first immunization, the mean OS associated with peptide vaccination was 8.3 months (3 to 21 months). All patients had died except for Patient P04. The survival rate was around 50% at 24 months according to **Table 3**. Clinical response of all patients in this study was shown in **Figure 2B**. Three out of seven patients showed tumor reduction at the target lesions. Patients P01 and P04 were evaluated as PR, as the sizes of their target lesions reduced by 54% and 57% respectively compared to those of baseline, while patient P06 maintained stable disease with the size of target lesion only reduced by 5%. Although different degrees of size increase at target lesions was observed for the other four patients (P02, P03, P05 and P07), patients P02, P03 and P07 were assessed as SD with less than 20% increase. Only patient P05 was evaluated as PD for a 40% increase in target lesion. The disease control rate (DCR) of the 7 patients was 85.71%.

**Table 3:** The survival and objective response rate of each patient.

Survival	Months
mOS	24.1
mPFS	3.1
mOS*	8.3
**Objective response rate**	**n/N**
CR	0/7
PR	2/7
SD	4/7
PD	1/7

mOS, mean Overall survival; mPFS, mean Progression-free survival; CR, Complete remission; PR, Partial response; SD, Stable disease; PD, Progressive disease;

*Calculated from the time the patient received the peptide vaccine.

### Case Report of Patient P01

**Figure 3:**
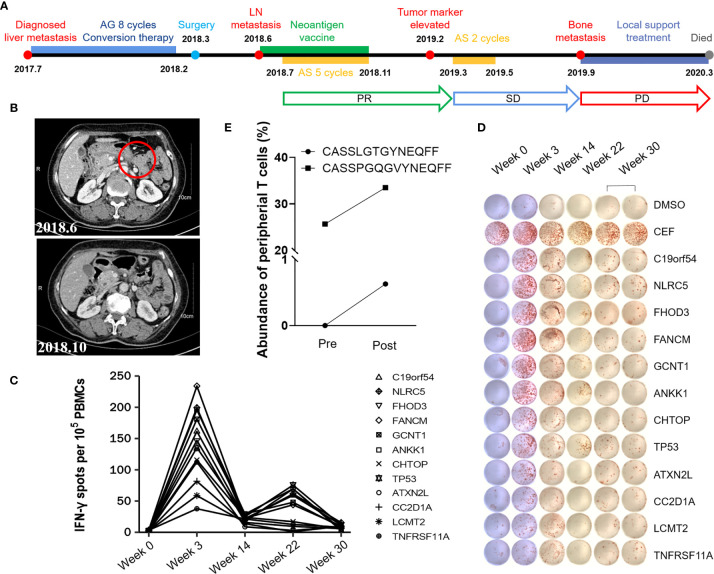
A case report of Patient P01. **(A)** Treatment timeline of P01. **(B)** Comparison of lymph node before & after vaccination by imaging. **(C, D)** *Ex vivo* IFN-γ ELISpot of PBMCs was performed with peptides at different time points. The dimethyl sulfoxide (DMSO) group was used as the negative control and mixed peptides from CEF (including peptides of cytomegalovirus, Epstein–Barr virus and influenza virus) were used as the positive control. **(E)** Increased abundance of peripheral T cell clones after vaccination was detected by TCR sequencing.

Patient P01 was first diagnosed with pancreatic cancer with liver metastasis in July 2017. From July 2017 to February 2018, she was given AG regimen (paclitaxel albumin plusS-1) for 8 cycles as first-line conversion chemotherapy. Under general anesthesia, laparoscopic radical pancreatectomy was performed on March 5th, 2018. No chemotherapy was given after surgery due to the patient’s poor physical condition. However, in less than three months, lymph node metastasis was found. From 6th June 2018 to 7th November 2018, Patient P01 received 8 doses of iNeo-Vac-P01 in total, including 5 prime and 3 boost immunizations. In addition, to maximize the patient’s clinical benefits, 5-cycle AS second-line treatment was given from July 2018 to November 2018 as a concomitant therapy. In February 2019, a tumor marker was detected while CT imaging showed no tumor recurrence. In response, the patient soon received 2-cycle AS systemic chemotherapy. After the treatment, the patient’s disease was considered to be well controlled until bone metastasis and pleural effusion occurred in September 2019. Since then, the disease progressed rapidly. After supportive treatment and chemotherapy, Patient P01 died in March 2020. The OS and PFS of Patient P01 were 32 and 8 months. It is noteworthy that the OS associated with iNeo-Vac-P01 treatment of Patient P01 was 21 months. The whole treatment regimen was shown in **Figure 3A**. MRI images showed the regression of pancreaticogastric nodule 4 months after first immunization when compared to baseline level (**Figure 3B**). No iNeo-Vac-P01-related serious AEs occurred during the whole vaccine treatment. *Ex vivo* IFN-γ ELISpot of PBMCs confirmed robust *de novo* immune response against all neoantigen peptides since vaccination, with a peak at Week 3 (**Figures 3C, D**). TCR sequencing of peripheral T cells revealed that the abundance of the TCR clone (CASSPGQGVYNEQFF) considerably increased after vaccination. Moreover, a new TCR clone (CASSLGTGYNEQFF) was detected after vaccination (**Figure 3E**). These data suggested that a subset of T cells with neoantigen specificities were induced by iNeo-Vac-P01 in Patient P01. However, no enough blood sample left could be applied to evaluate whether these TCR clones recognize the same peptide. The concomitant iNeo-Vac-P01 therapy and chemotherapy might have generated synergetic benefits to prolong the OS and PFS of Patient P01.

### Immune Response

**Figure 4:**
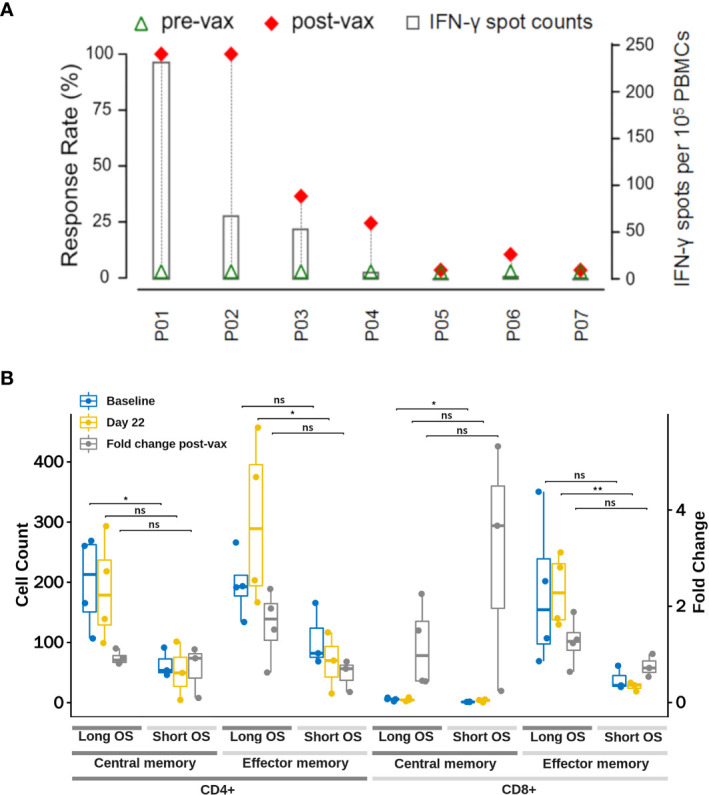
Immune Response induced by iNeo-Vac-P01. **(A)** iNeo-Vac-P01 induced specific T cell response. For each patient marked in X-axis, green triangle and red diamond represent the response rates pre- and post-vaccination, defined as the ratios of the numbers of peptides (or peptide pools) with positive ELISpot results before and after vaccination to the total number of peptides (or peptide pools) in vaccine, respectively. The bar chart with secondary Y-axis represented the IFN-γ spots per 10^5^ PBMCs of the peptide with best response for each patient. **(B)** Change in T cell subsets count at baseline and 22 days post iNeo-Vac-P01 vaccination. Blue and yellow dots represent the cell counts at baseline and 22 days post iNeo-Vac-P01, respectively. Grey dots represent the fold change of corresponding cell count, which can be read at the right Y-axis. Counts of T cell subsets including CD4^+^ central memory, CD4^+^ effector memory, CD8^+^ central memory and CD8^+^ effector memory T cells were compared in long OS patients (shown in dark grey) and short OS patients (shown in light grey). *: significant, p=0.01 to 0.05; **: very significant, p=0.001 to 0.01; ns: not significant.

**Figure 5:**
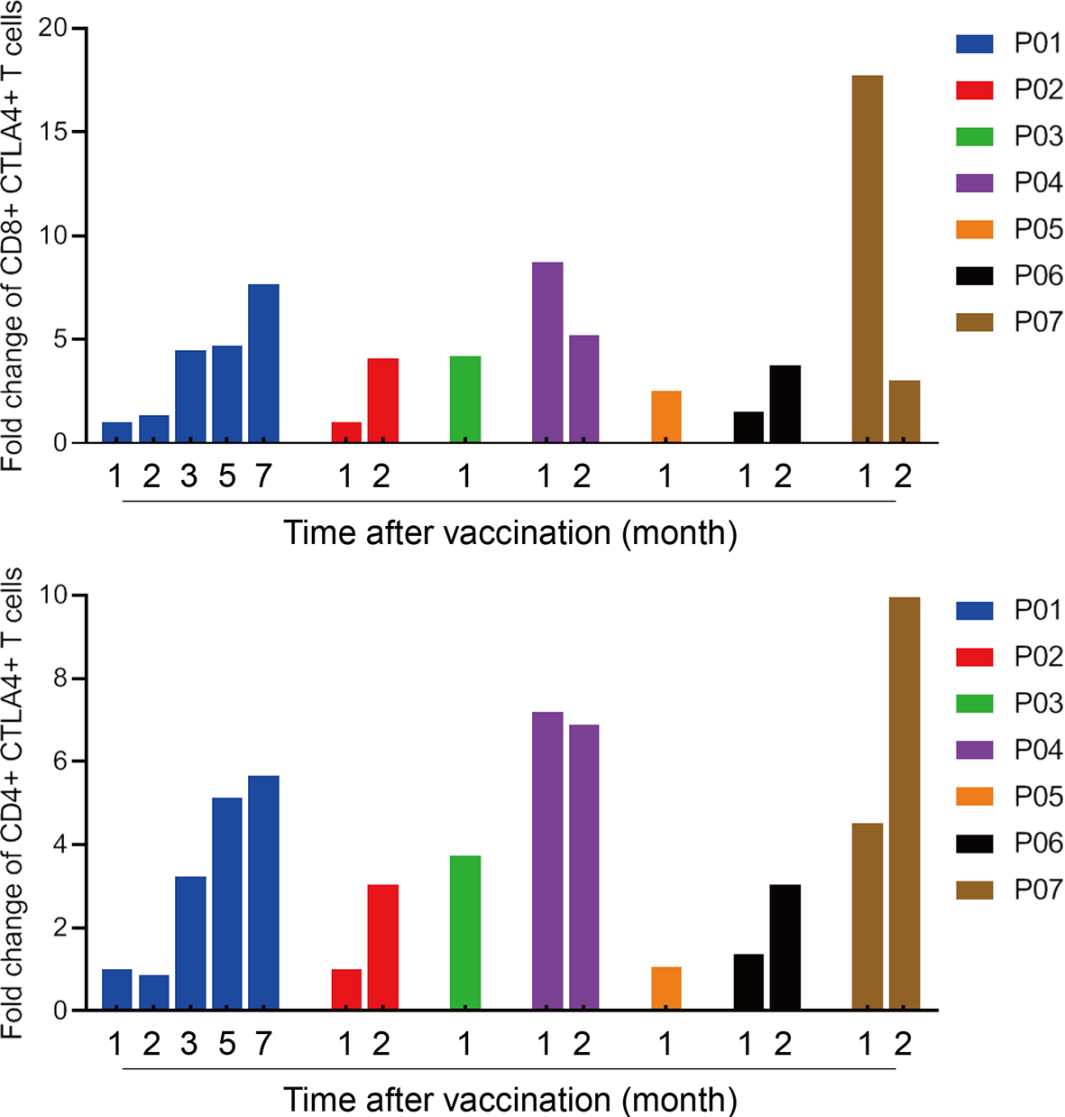
The proportion of CTLA4+ T cells in peripheral blood increased after vaccination. The proportions of CD8+ CTLA-4+ T cells and CD4+ CTLA-4+ T cells ratio to total T cells in peripheral blood were determined by flow cytometry. Fold changes of post vaccination compared with baseline were calculated.

*Ex vivo* IFN-γ ELISpot assay was performed with autologous PBMCs after vaccination. ELISpot assay results demonstrated the potentials of iNeo-Vac-P01 to induce the activation of T cells in 5 out of 7 (71.4%) patients (**Figure 4A** and **Supplementary Table 6**). For each patient, the number of IFN-γ spots per 10^5^ PBMCs of the peptide or peptide pool with best response was shown in **Figure 4A**. For patients P05 and P07, no evident response was found pre- and post-vaccination. Overall, 31 out of 70 (44.3%) individual long peptides elicited measurable peptide-specific immune responses (positive results in ELISpot assay after vaccination). No correlation between IL-2, IL-6, IL-10, TNF-α and clinical response was found, as the titers of these cytokines did not change drastically after vaccination. However, for patients who had a relatively long OS (P01, P04 and P07), their IFN-γ titer in the peripheral blood increased to a much larger extent after vaccination, compared to patients who had relatively short OS (P03, P05 and P06) (**Supplementary Table 7**). This phenomenon suggested that IFN-γ titer in the peripheral blood could be a potential biomarker for clinical response. It is noted that not every patient managed to provide sufficient blood sample for cytokine studies due to their poor physical conditions at the designated time points (P04). Moreover, for all 7 patients, the proportions of CD4+ CTLA4+ T cells and CD8+ CTLA4+ T cells in peripheral blood increased during vaccination (**Figure 5**), suggesting that combined treatment with anti-CTLA-4 immunotherapy might help achieve stronger antitumoral immune response. It is noteworthy that not all patients provided enough blood samples for analysis due to their poor physical conditions at the designated time points (e.g. P03 and P05). In addition, changes in the T cell subsets post vaccination also suggested that CD4^+^ or CD8^+^ effector memory T cells in the peripheral blood could be a potential biomarker for clinical response. Before treatment, patients with relatively long OS (P01, P02, P04 and P07) had more CD4^+^ or CD8^+^ central memory T cells (T_CM_) and CD4^+^ or CD8^+^ effector memory T cells (T_EM_) than patients with relatively short OS (P03, P05 and P06); however, only the difference in T_CM_ showed statistical significance (p<0.05). Post vaccination, longer OS patients still had more T_CM_ and T_EM_, while only T_EM_ showed statistical significance (p<0.05) (**Figure 4B** and **Supplementary Table 8**). This phenomenon indicated that upon antigenic stimulation, differentiation of central memory T cells to effector memory T cells might be activated (30).

## Discussion

In this study, we retrospectively analyzed the safety and tolerability of neoantigen-based peptide vaccine iNeo-Vac-P01 in pancreatic cancer patients. Although mRNA vaccines have gained much research interest since the approval of mRNA-based COVID-19 vaccines under Emergency Use Authorization for COVID-19, no mRNA therapy was approved during the time this study was conducted. Thus, well-investigated peptide-based vaccine approach was used in this study. None of the patients enrolled in this study showed SAE during vaccination, while only one patient showed vaccine-related AE (slight rash) which recovered without any nursing.

Currently, the median overall survival time for advanced pancreatic cancer patients is only 6 to 9 months (31). Albumin paclitaxel combined with gemcitabine or mFOLFIRINOX regimen [5-fluorouracil (5-FU), irinotecan and oxaliplatin] is recommended as the standard first-line treatment by the guidelines (32, 33). Meanwhile, there is no standard chemotherapy regimen after second-line treatment. A stratified analysis of the posterior survival of pancreatic cancer patients showed that the median survival time of patients with high-risk pancreatic cancer was only 1.4 months, and that of low-risk patients was less than 12 months (34). To our knowledge, advanced pancreatic cancer patients tend to have larger tumor burdens than patients diagnosed with other cancer types, therefore, instead of applying monotherapy of neoantigen vaccination, chemotherapy or ICI (i.e. anti-PD-1) therapy were scheduled to several patients (P01 and P07) to maximize the clinical benefits for them. The treatment regimen for each patient was listed in **Supplementary Table 1**. The mean OS and PFS of advanced pancreatic cancer patients in this retrospective study were 24.1 and 3.1 months, longer than most of the clinical data reported in other clinical studies. In addition, the mean OS associated with the vaccine treatment was 8.3 months. Although this retrospective study has a relatively small sample size, the results here demonstrated promising potentials of using personalized neoantigen-based peptide vaccine as a second-line or later treatment to prolong the survival duration of an advanced pancreatic cancer patient.

GM-CSF was used as a molecular adjuvant for personalized vaccine in this study due to ethical concerns, as choices of approved vaccine adjuvants in China were limited. No GM-CSF control group was set since this study was a retrospective cohort study with a small sample size, instead of a randomized controlled trial. However, in future phase 2 study, GM-CSF could be used as control.

KRAS mutation is a common driver mutation for several cancer types including pancreatic cancer. It is usually incorporated in the “shared” tumor antigen combinations as “off-the-shelf” tumor vaccines. In a study of patients with advanced cancer (pancreatic cancer, cholangiocarcinoma and colorectal cancer), Rahma et al. demonstrated that the patients could achieve a mean PFS and OS of 3.6 months and 16.9 months respectively, with an immune response rate of 54% when treated with the vaccine formulation containing KRAS G12D, G12V and G12C peptides (35). Although it is a promising strategy to treat the patients with KRAS mutations, the “off-the-shelf” tumor vaccines cannot fulfill the clinical needs for those who do not harbor the mutations. Different from the vaccines based on “shared” antigens, personalized neoantigen-based peptide cancer vaccines are customized for each patient. In this retrospective study, only 5 out of 7 patients had KRAS mutations (**Supplementary Table 9**). KRAS mutations were also included in the design of personalized iNeo-Vac-P01 for these 5 patients to maximize the clinical benefits. For Patient P01 who did not have KRAS mutation had a significant extension of survival duration as a result of concomitant iNeo-Vac-P01 vaccine therapy with chemotherapy (**Figure 3** and **Table 1**).

It is also noteworthy that Patient P01 started the personalized peptide vaccination only three months after the primary tumor resection, leading to relatively lower tumor burden compared to other patients. The iNeo-Vac-P01-related OS of Patient P01 was 21 months which was longer than that of any other patients in this study. More studies should be conducted in the future to investigate whether a pancreatic cancer patient could achieve longer survival duration if neoantigen tumor vaccine is given when the tumor burden is low. Previously, a case report had described a pancreatic cancer patient who began SVN-2B peptide vaccine treatment during the adjuvant treatment stage (20). After the vaccine regimen, isolated lung metastases were observed in this patient but subsequently well controlled by surgery. It was reported that this patient had a survival duration of more than 10 years (20). In light of this case report together with our clinical findings with Patient P01, we believe that it is important to investigate the “perfect” timing for the administration of neoantigen tumor vaccine, for instance, when the tumor burden is low.

In addition, several T cell subsets and cytokines in peripheral blood were evaluated in this study to identify potential biomarkers for clinical response. The drastic fold-change of IFN-γ titer in peripheral blood observed in patients with relatively long OS (P01, P02 and P07), in comparison with patients with relatively short OS that did not experience significant IFN-γ change (P03, P05 and P06), has suggested that peripheral IFN-γ titer could be a biomarker for clinical response. Interestingly, these patients with short OS happened to have very short vaccine-associated OS as well. Although P02 and P07 had relatively long OS compared to P03, P05 and P06, the vaccine-associated OS for them was short. This could be attributed to the late administration of vaccines for them, which again, emphasizes the point of interventional vaccination scheduled at an earlier time point after first-line or second-line chemotherapy can be more beneficial to achieve better clinical response. As a result of induction by peptide vaccines, both CD4+ CTLA4+ T cell and CD8+ CTLA4+ T cell populations in peripheral blood had increased in all 7 patients. Therefore, combined treatment of personalized neoantigen-based peptide vaccines with anti-CTLA-4 antibody could potentially be a promising treatment modality for pancreatic cancer patients. A shift from CD4^+^ or CD8^+^ T_CM_ to CD4^+^ or CD8^+^ T_EM_ was observed in long OS patients (P01, P02, P04 and P07) upon vaccination, showing significant differences with relatively short OS patients (P03, P05 and P06). This indicated the successfully antigenic stimulation that led to the differentiation of T_CM_ to T_EM_. In all, further studies with special considerations of the time for neoantigen vaccine administration should be conducted to achieve better clinical benefits for cancer patients such as pancreatic cancer patients.

## Conclusions

This retrospective study demonstrated the feasibility of neoantigen selection for pancreatic cancer patients with low TMB (less than 10 mutations/Mb), as well as the tolerability of personalized neoantigen-based peptide vaccine, iNeo-Vac-P01, for treating pancreatic cancer. Our findings were important and complementary to previously published studies in neoantigen cancer vaccines treating other types of cancers. The development and implementation of personalized neoantigen-based peptide cancer vaccines might provide a new strategy to improve the limited clinical efficacy of traditional treatments for pancreatic cancer.

## Data Availability Statement

The datasets presented in this study can be found in online repositories. The names of the repository/repositories and accession number(s) can be found below: https://ngdc.cncb.ac.cn/gsa-human/s/UHnoE84R.

## Ethics Statement

The studies involving human participants were reviewed and approved by Ethics Committee of Zhejiang Provincial People’s Hospital. The patients/participants provided their written informed consent to participate in this study.

## Author Contributions

ZC, SZ, SC, LY, and FM contributed to the conception and design of the work. ZC, NH, DM and XG conducted the studies. JJ, YX, HW, and MQ contributed to data processing and analysis. ZC, QH, FM, and NH helped with the statistical analysis. ZC, SZ, LL, SC, and LY prepared most part of the manuscript with FM. NH revised it critically. All authors contributed to the article and approved the submitted version.

## Funding

This work was supported by National Natural Science Foundation of China (U20A20409 to SC, 81772575 and 81972455 to Liu Yang, and 81802623 to ZC).

## Conflict of Interest

FM is an employee for Hangzhou Neoantigen Therapeutics Co., Ltd and Hangzhou Al-Force Therapeutics. SZ, NH, DM, HW, XG, KL, MQ, QH, LL, and SC are employees for Hangzhou Neoantigen Therapeutics Co., Ltd.

The remaining authors declare that the research was conducted in the absence of any commercial or financial relationships that could be construed as a potential conflict of interest.

## Publisher’s Note

All claims expressed in this article are solely those of the authors and do not necessarily represent those of their affiliated organizations, or those of the publisher, the editors and the reviewers. Any product that may be evaluated in this article, or claim that may be made by its manufacturer, is not guaranteed or endorsed by the publisher.
