# Supplementary material for: A Neoantigen-Based Peptide Vaccine for Patients With Advanced Pancreatic Cancer Refractory to Standard Treatment
Source: Front Immunol. 2021 Aug 13;12:691605. doi: 10.3389/fimmu.2021.691605 (PMC8414362; doi:10.3389/fimmu.2021.691605)
Supplement: Supplementary file 1 [file DataSheet_1.pdf]

Supplementary Materials

Supplemental Information for Methods

Generation of neoantigen peptides

Tumor neoantigens were predicted and prioritized by in-house bioinformatics pipeline iNeo-SUITE, which consists modules of sequencing read filtering, genome alignment, mutation calling, HLA typing, MHC affinity prediction, gene expression profiling, vaccine peptide sequence design and prioritization based on therapeutic potency. FastQC (v0.11.4) was used for sequencing data quality control. Reads with quality score below 15 or more than 4 N bases were discarded [1] . The qualified reads were then mapped to the human reference HG38 (Human Genome version 38) by using Burrows-Wheeler Aligner software (BWA, v0.7.12). Next, comparing to normal sample, tumor somatic mutations were identified by integrating the mutation calling results from Mutect (v2.0), Varscan 2 (v.3.5.19) [2-5], Strelka (1.0.11) and somatic-sniper (v1.0.5.0). Then, somatic mutation candidates were ranked based on their reliability, and were manually inspected by Integrative Genomics Viewer (IGV v.1.0.6) according to their alignment profile. Meanwhile, germline mutations in both normal and tumor samples were identified by GATK HaplotypeCaller. The database of Single Nucleotide Polymorphism (dbSNP) (https://www.ncbi.nlm.nih.gov/snp/) and 1000 Genome datasets were used to filter out high population frequency (PF) mutations (PF > 1%) from somatic mutation candidates. Next, the mutations were further annotated by Variation Effect Predictor (VEP, ensemble v89) [5-7]. Using the reference sequences from IMGT datasets [6], HLA typing and quantification was done by OptiType (v1.3.1), Polysolver (V4), PHLAT (release 1.1) and in-house software iNeo-HLA. Subsequently, the flanking sequence of peptide or the upstream sequence of peptide were extracted from human protein database for single nucleotide mutations or frameshift and stop-loss mutations. To predict the neo-epitopes within those peptides, all possible segments that contain mutation-induced amino acid(s) were further extracted with length ranging from 8 to 16

amino acids (8-11 mer for HLA class I and 12-16 mer for HLA class II). The HLA class I neo-epitopes were predicted by in-house software iNeo-Pred, a deep-learning and machine-learning integrated predictor trained on datasets from IEDB and mass spectrometry (MS) profiling of HLA ligands. An epitope was considered to have binding affinity if the result from iNeo-Pred was below 500 nM. The HLA class II neo-epitopes were predicted by NetMHCIIPan (v4.0) according to the manual from official website [8].

After identifying all the neo-epitope candidates, in-house software iNeo-PRIOR was used to rank mutations based on their therapeutic potency, whereas mutation prevalence, gene expression, affinity change, epitope number, and heterologous level of mutant peptide, etc. were taken into consideration. Since all these factors contribute to the final therapeutic effect, a mathematical formula was designed to integrate all these factors into a single score for prioritization:

$$iNeo\_Score = \mathrm{f}_1(\mathrm{Ag}) \times \mathrm{f}_2(\mathrm{E}) \times \mathrm{f}_3(\mathrm{M_i}) \times \mathrm{f}_4(\mathrm{H}) + \mathrm{f}_5(\mathrm{M_{ii}})$$

In this formula, *iNeo_Score* refers to the score for prioritization, Ag stands for mutation prevalence, E stands for the average gene expression obtained from TCGA database, and H stands for the heterologous level of mutant peptide. M_i and M_{ii} stand for the quality index which take affinity change and epitope number into account for epitopes presented by MHC I molecules and MHC II molecules respectively. Mutations with top ranking scores were subjected for choosing, while other factors such as the reliability of the mutation (assessed by manual examination and Sanger sequencing) and gene function (whether the mutation was in an oncogenic or cancer-driver gene) were also considered.

In-house software iNeo-DESIGN was applied to automatically designed vaccine peptide sequences (length ranging from 15 to 30 amino acids) containing neo-epitopes of both HLA class I and II. Safety issues such as potential peptide toxicity and bioactivity, as well as the difficulties in peptide synthesis were evaluated and optimized accordingly. Finally, the

customized long peptides were manufactured by chemical synthesis at GMP-like standard and clinical-grade (bacteria-free, >95.0% purity with less than 10 EU/mg bacterial endotoxin).

IFN-γ enzyme-linked immunospot (ELISpot) assay

Peripheral blood (10-30 mL) was collected from each patient, followed by the isolation of peripheral blood mononuclear cells (PBMCs) by Ficoll/Hypaque density-gradient centrifugation (GE Healthcare). IFN-γ ELISpot assays were performed with Human IFN-γ precoated ELISpot kit (DAKEWEI). Briefly, after adding 200μL serum-free medium into each well, the plate was incubated at room temperature for 5-10 minutes before discarding the solution. 100 μL cell suspension was added to each well at a density of 2×10^5 cells per well, followed by the addition of 5-10 μg/mL neoantigen peptide into the same well as sample or 2 μg/mL of CEF peptide as positive control. Then the mixtures were incubated at 37 °C for 16-24 hours. 200 μL pre-cooled deionized water was added into each well to lyse at 4 °C for 10 minutes. The plates were washed 6 times before the addition of 100 μL biotin-labeled antibody and then incubated at 37 °C for 1 hour. After washing the plates, 100 μL enzyme-labeled avidin working solution was added into each well and incubated at 37 °C for 1 hour. AEC solution mix was then added into each well after washing the plates, and the plates were kept in the dark for 25 minutes at room temperature before adding deionized water to stop the reaction. ELISpot plate was then placed in an automatic plate reader set with appropriate parameters, spot count and statistical analysis. The samples with more than 100 spots after noise subtraction (based on negative control group) were considered to show strong positive results, while samples with 8 to 20 spots were considered to show weak positive results.

Cytometric analysis of T-lymphocyte activity through surface biomarker

Antibodies were purchased from Biolegend, as shown in Table A. PBMCs were isolated, and T cells were labeled following manual instruction. In brief, the corresponding antibodies were added into an empty flow tube, mixed with 100 μL T cell sample thoroughly, and then incubated

in the dark for 15 minutes. 2 mL of erythrocyte lysate (Zhejiang Bozhen Biotechnology Co., Ltd.) was added into the sample, mixed entirely, and then incubated in the dark for 10 minutes. The sample was centrifuged at 500×g for 5 minutes, and 1620 μL of supernatant (440 μL remained) was removed. Next, 10 μL of absolute count microspheres was added into the tube and mixed well. Cytometric analysis was conducted after sample preparation.

Table A: Antibodies for flow cytometry

Fluorescence	Antibody	Clone
FITC	CD279（PD-1）	EH12.2H7
PE	CD197（CCR7）	G043H7
PerCP/Cy5.5	CD4	OKT4
PE/Cy7	CD45RA	HI100
APC	CD38	HB-7
A700	CD8	SK1
APC/Cy7	CD3	
BV421	HLA-DR	
BV510	CD45	
BV605	CD152（CTLA-4）	BNI3

Cytometric Bead Array (CBA) Analysis of Cytokines

The concentrations of serum cytokines were measured by CBA, according to the manufacture's protocol (Hangzhou Saiji Biotechnology Co., Ltd). Th1/Th2 cytokine kit was applied. In brief, 25

μL solution of captured microspheres was added into a blank flow tube, followed by the addition of 25 μL buffer solution of microspheres. The mixture was incubated in the dark for 30 minutes. 25 μL fluorescence detection reagent and 25 μL serum were added successively. The solution was vortex-mixed and then incubated in the dark for 2.5 hours. After the addition of 1mL of PBS solution, the sample was centrifuged at 200×g for 5 minutes. Following the removal of supernatant, 100 μL PBS solution was added to resuspend the sample. The samples were tested by a flow cytometer, and the acquired data were analyzed using FlowJo V10 software.

References

1. Hundal, J., et al., *Cancer Immunogenomics: Computational Neoantigen Identification and Vaccine Design.* Cold Spring Harb Symp Quant Biol, 2016. **81**: p. 105-111.

2. Chen, F., et al., *Neoantigen identification strategies enable personalized immunotherapy in refractory solid tumors.* J Clin Invest, 2019. **130**.

3. Hundal, J., et al., *pVAC-Seq: A genome-guided in silico approach to identifying tumor neoantigens.* Genome Med, 2016. **8**(1): p. 11.

4. Ott, P.A., et al., *An immunogenic personal neoantigen vaccine for patients with melanoma.* Nature, 2017. **547**(7662): p. 217-221.

5. Ng, A.W.R., et al., *In silico-guided sequence modifications of K-ras epitopes improve immunological outcome against G12V and G13D mutant KRAS antigens.* PeerJ, 2018. **6**: p. e5056.

6. Hilf, N., et al., *Actively personalized vaccination trial for newly diagnosed glioblastoma.* Nature, 2019. **565**(7738): p. 240-245.

7. Keskin, D.B., et al., *Neoantigen vaccine generates intratumoral T cell responses in phase Ib glioblastoma trial.* Nature, 2019. **565**(7738): p. 234-239.

8. Andreatta, M., et al., *Accurate pan-specific prediction of peptide-MHC class II binding affinity with improved binding core identification.* Immunogenetics, 2015. **67**(11-12): p. 641-50.

Supplementary Tables

Supplementary Table 1. The treatment process for patients before and after neoantigen vaccine therapy

Supplementary Table 2. QC metrics of whole-exome sequencing for each patient

Supplementary Table 3. Summary of the number of identified somatic mutations, predicted neoantigens and synthesized vaccine peptides

Supplementary Table 4. HLA allotypes in both normal and tumor cells for each patient

Supplementary Table 5. Summary of designed and synthesized peptides for each patient

Supplementary Table 6. The best response of each peptide/peptide pool for all 7 patients

Supplementary Table 7. Cytokine titers in peripheral blood for each patient

Supplementary Table 8. T-cell subsets before and after vaccination

Supplementary Table 9. Mutation of KRAS for each patient

Supplementary Table S1. The treatment process for patients before and after neoantigen vaccine therapy

Patients	Diagnosed time	Treatment before enrollment					Neoantigen peptide vaccine treatment	Combination chemotherapy	Late follow-up and assessment
		Conversion therapy	Surgery	First-line chemotherapy	Second-line chemotherapy	Others			
P01	July,2017	July 2017 to January 2018, AS 8 cycles	March 2018，Laparoscopic radical resection of pancreatic cancer	/	/	/	June 2018 to November 2018	July 2018 to November 2018, AS 5 cycles	For a period of time after the trial, the patient's disease was well controlled; pleural effusion occurred in September 2019, and the disease progressed rapidly. After supportive treatment and chemotherapy, she died in March 2020
P02	April,2016	May 2016 to July 2016, AG 3 cycles	August 2016, pancreatococcygeal combined splenectomy	August 2016 to October 2017, AG 3 cycles+ GS 11 cycles.	November 2017 to May 2018, FOLFIRINOX 10 cycles	/	June 2018 to August 2018	/	After progression, the patients left the group and were treated with chemotherapy locally. The specific situation is unknown.The patient died of progressive disease on December 31, 2018
P03	December,2017	/	December 2017,Laparoscopic radical resection of pancreatic cancer	NA	/	/	October 2018 to February 2019	/	Died in February 2019
P04	October,2017	October 2017 to February 2018, FOLFIRINOX 8 cycles	Patient refused	February 2018 to April 2018, FOLFIRINOX 3 cycles	October 2018 to November 2018, GEMOX 2 cycles,the patients could not tolerate the chemotherapy	/	December,2018 to February 2019	/	The patient was treated locally and died in April 2020 due to tumor progression and biliary obstruction
P05	August, 2017	August 2017 to October 2017, AG 2 cycles;October 2017 to December 2017 GS 3 cycles	Patient refused	December 2017 to February 2018, GS 2 cycles	February 2017 to May 2018, FOLFIRINOX 7 cycles	/	September 2018 to October 2018	/	Died in January 2019
P06	November,2017	/	December 2017, laparoscopic pancreaticoduodenectomy	February 2018 to June 2018, AG 5 cycles，the patients could not tolerate the chemotherapy	/	/	July 2018 to September 2018	/	Died in November 2018
P07	December,2017	/	January 2018，Laparoscopic radical resection of pancreatic cancer	January 2018 to May 2018, AG 4 cycles	June 2018 to December 2018, FOLFIRINOX 13 cycles;From December 2018, the PD-1 inhibitor was combined.	March 2019 to June 2019,AS plus PD-1 inhibitor 5 cycles	August 2019 to October 2019	September 2019, PD-1 inhibitor 1 cycle	Gastric pyloric obstruction on October 2019. Died in January 2020.

FOLFIRINOX,5-fluorouracil [5-FU], irinotecan, and oxaliplatin;AS, Albumin paclitaxel plus S-1; AG, Albumin paclitaxel plus gemcitabine; GEMOX, oxaliplatin plus gemcitabine; GS, gemcitabine plus S-1

Supplementary Table S2. QC metrics of whole-exome sequencing for each patient

	P01		P02		P03		P04		P05		P06		P07	
	Normal	Tumor	Normal	Tumor	Normal	Tumor	Normal	Tumor	Normal	Tumor	Normal	Tumor	Normal	Tumor
Q30%	95.11%	86.56%	93.20%	92.27%	94.47%	89.78%	93.79%	90.24%	94.14%	86.37%	94.47%	88.21%	95.60%	95.52%
[Total] Raw Reads (All reads)	136986313	456410163	73972329	203516471	96941684	328345200	69039548	232823532	90938597	395302256	106480675	207874214	211301468	210937507
[Target] Average depth	275.32	669.35	121.35	337.34	201.61	583.3	141.41	342.2	170.31	639.39	166.24	233.15	387.58	381.55
[Target] Coverage (>=30x)	97.80%	99.59%	97.15%	99.59%	98.57%	99.75%	97.40%	99.05%	98.23%	99.75%	94.77%	91.94%	99.54%	99.30%
[Target] No Coverage	0.21%	0.35%	0.06%	0.04%	0.17%	0.12%	0.08%	0.03%	0.05%	0.02%	1.90%	1.40%	0.18%	0.33%
Contamination	0.12%	8.20%	0.07%	0.10%	0.00%	0.46%	0.23%	2.29%	0.13%	2.23%	0.24%	3.80%	0.09%	0.08%
Tumor Purity (%)	0.647		0.32%		0.353		0.374		0.28		0.72		0.38	

Supplementary Table S3. Summary of the number of identified somatic mutations, predicted neoantigens and synthesized vaccine peptides

Patient ID		P01	P02	P03	P04	P05	P06	P07
Total mutations	Total number	82340	169	3330	11815	8688	5394	114
	Missense_Mutation	33663	104	2045	7404	5613	3335	96
	In_Frame_InDel	474	7	17	249	51	40	14
	Frame_Shift_InDel	566	4	53	360	55	111	3
	Nonstop_Mutation	104	0	33	51	20	41	1
	Other	47533	54	1182	3751	2949	1867	0
Mutations with more than one neoantigen	Total number	1808	111	2131	4957	5711	3493	104
	Missense_Mutation	1751	103	2077	4615	5598	3316	96
	In_Frame_InDel	22	5	14	135	41	34	8
	Frame_Shift_InDel	31	3	40	179	52	105	0
	Nonstop_Mutation	4	0	0	28	20	38	0
Predicted epitopes	Class I	24800	1082	38792	66616	69884	54049	760
	Class II	71175	3957	152515	189842	233565	229590	766
Number of peptides included in vaccine		12	11	12	13	4	13	5

Supplementary Table S4. HLA allotypes in both normal and tumor cells for each patient

Patient ID	Normal							
	HLA-class I			HLA-class II				
	HLA-A	HLA-B	HLA-C	HLA-DQA1	HLA-DQB1	HLA-DPA1	HLA-DPB1	HLA-DRB1
P01	HLA-A30:01	HLA-B40:01	HLA-C06:02	HLA-DQA1*02:01	HLA-DQB1*02:02	HLA-DPA1*02:01	HLA-DPB1*05:01	HLA-DRB1*07:01
	HLA-A02:03	HLA-B13:02	HLA-C04:03	HLA-DQA1*06:01	HLA-DQB1*03:01	HLA-DPA1*02:02	HLA-DPB1*17:01	HLA-DRB1*12:02
	-	-	-	-	-	-	-	-
P02	HLA-A24:02	HLA-B40:01	HLA-C03:03	HLA-DQA1*03:02	HLA-DQB1*03:01	HLA-DPA1*02:02	HLA-DPB1*02:02	HLA-DRB1*09:01
	HLA-A11:01	HLA-B13:01	HLA-C03:04	HLA-DQA1*05:05	HLA-DQB1*03:03	-	HLA-DPB1*19:01	HLA-DRB1*12:01
	-	-	-	HLA-DQA1*05:08	-	-	-	-
P03	HLA-A02:03	HLA-B39:01	HLA-C08:01	HLA-DQA1*01:03	HLA-DQB1*03:01	-	-	HLA-DRB1*08:03
	HLA-A11:01	HLA-B15:02	HLA-C07:02	HLA-DQA1*06:01	HLA-DQB1*06:01	-	-	HLA-DRB1*12:02
P04	HLA-A02:01	HLA-B15:07	HLA-C03:03	HLA-DQA1*03:02	HLA-DQB1*03:03	HLA-DPA1*02:02	HLA-DPB1*05:01	HLA-DRB1*09:01
	HLA-A11:01	HLA-B46:01	HLA-C01:02	-	-	-	-	-
P05	HLA-A02:07	HLA-B40:01	HLA-C01:02	HLA-DQA1*01:03	HLA-DQB1*03:01	HLA-DPA1*02:02	HLA-DPB1*02:02	HLA-DRB1*08:03
	HLA-A11:01	HLA-B46:01	HLA-C07:02	HLA-DQA1*06:01	HLA-DQB1*06:01	-	HLA-DPB1*05:01	HLA-DRB1*12:02
P06	HLA-A02:03	HLA-B13:01	HLA-C03:04	HLA-DQA1*01:02	HLA-DQB1*05:02	HLA-DPA1*01:03	HLA-DPB1*05:01	HLA-DRB1*12:02
	HLA-A11:01	HLA-B40:01	HLA-C04:03	-	HLA-DQB1*06:01	HLA-DPA1*02:02	HLA-DPB1*21:01	HLA-DRB1*15:01
P07	HLA-A26:01	HLA-B40:01	HLA-C01:04	-	-	-	-	HLA-DRB1*03:17
	HLA-A01:02	HLA-B07:03	HLA-C07:02	-	-	-	-	HLA-DRB1*11:30

Patient ID	Tumor							
	HLA-class I			HLA-class II				
	HLA-A	HLA-B	HLA-C	HLA-DQA1	HLA-DQB1	HLA-DPA1	HLA-DPB1	HLA-DRB1
P01	HLA-A02:03	HLA-B13:02	HLA-C04:03	HLA-DQA1*02:01	HLA-DQB1*02:02	HLA-DPA1*02:01	HLA-DPB1*05:01	HLA-DRB1*07:01
	HLA-A30:01	HLA-B40:01	HLA-C06:02	HLA-DQA1*06:01	HLA-DQB1*03:01	HLA-DPA1*02:02	HLA-DPB1*17:01	HLA-DRB1*12:01
	-	-	-	-	-	-	-	HLA-DRB1*12:02
P02	HLA-A24:02	HLA-B13:01	HLA-C03:03	HLA-DQA1*03:02	HLA-DQB1*03:01	HLA-DPA1*02:02	HLA-DPB1*02:02	HLA-DRB1*09:01
	HLA-A11:01	HLA-B40:01	HLA-C03:04	HLA-DQA1*05:05	HLA-DQB1*03:03	-	HLA-DPB1*19:01	HLA-DRB1*12:01
	-	-	-	HLA-DQA1*05:08	-	-	-	-
P03	HLA-A02:03	HLA-B39:01	HLA-C08:01	HLA-DQA1*01:03	HLA-DQB1*03:01	-	-	HLA-DRB1*08:03
	HLA-A11:01	HLA-B15:02	HLA-C07:02	HLA-DQA1*06:01	HLA-DQB1*06:01	-	-	HLA-DRB1*12:02
P04	HLA-A02:01	HLA-B15:07	HLA-C01:02	HLA-DQA1*03:02	HLA-DQB1*03:03	HLA-DPA1*02:02	HLA-DPB1*05:01	HLA-DRB1*09:01
	HLA-A11:01	HLA-B15:01	HLA-C03:03	-	-	-	-	-
P05	HLA-A02:07	HLA-B40:01	HLA-C01:02	HLA-DQA1*01:03	HLA-DQB1*03:01	HLA-DPA1*02:02	HLA-DPB1*02:02	HLA-DRB1*08:03
	HLA-A11:01	HLA-B46:01	HLA-C07:02	HLA-DQA1*06:01	HLA-DQB1*06:01	-	HLA-DPB1*05:01	HLA-DRB1*12:02
P06	HLA-A02:51	HLA-B13:01	HLA-C03:04	HLA-DQA1*01:02	HLA-DQB1*05:02	HLA-DPA1*01:03	HLA-DPB1*05:01	HLA-DRB1*12:02
	HLA-A11:01	HLA-B40:01	HLA-C04:03	-	HLA-DQB1*06:01	-	HLA-DPB1*21:01	HLA-DRB1*15:01
P07	HLA-A26:01	HLA-B40:01	HLA-C01:04	HLA-DQA1*03:01	-	-	-	HLA-DRB1*03:17
	HLA-A01:02	HLA-B07:03	HLA-C07:02	HLA-DQA1*04:01	-	-	-	HLA-DRB1*11:30

Supplementary Table 5. Summary of designed and synthesized peptides for each patient

Patient ID	Immunizing pools	Peptide_ID	Gene_name	Chr	POSITION	Mutation_type	Designed_peptide_for_synthesis	Tumor Variant Freq.(exome)	Number of Class I epitopes	Number of Class II epitopes	Water solubility
P01	C	2	C19orf54	chr19	41255500	SNV	LPLPPPPAATGPAPPHVFGLEKSQLLKEAF	0.203	6	43	Yes
P01	A	3	NLRC5	chr16	57060371	SNV	HSLLTSFCVCTGPGHKQTGYAFTHLSLQEFLKK	0.150	8	15	Yes
P01	C	5	FHOD3	chr18	34324091	SNV	TVHKQSLLHHVCTMVIENFPDSSDLY	0.140	13	35	Yes
P01	B	6	FANCM	chr14	45623981	SNV	EDFMKLYNHLECMFAHTRSTSANGISAIQQG	0.125	16	58	Yes
P01	C	7	GCNT1	chr9	79117751	SNV	DRLLRAIYMPQNFYCVHVDTKSE	0.120	20	27	Yes
P01	A	9	ANKK1	chr11	113270015	SNV	KDGWAPLHFAAQNGDDRTARLLLDHGACVDAQ	0.100	7	20	Yes
P01	B	10	CHTOP	chr1	153609068	SNV	MSAQSAPKVVLKSTTKM	0.100	3	2	Yes
P01	B	12	TP53	chr17	7577558	Frameshift deletion	KKKKKCTTIHYNYMCNSSAWAA	0.100	15	13	Yes
P01	-	13	FBXO25	chr8	363192	SNV	CSQKLERENNHCNISHSIILNSEDGEKK	0.097	20	22	No
P01	-	14	ANK2	chr4	114095671	SNV	VVEYLKGGIDINTCNKNGLNALHLAAKEGHV	0.090	13	27	No
P01	A	15	ATXN2L	chr16	28847378	SNV	HPPQSHGGPPQGAVPPSGVPALSASTPSPYP	0.090	5	56	Yes
P01	B	16	CC2D1A	chr19	14020728	SNV	KKKKDGANDEELEAEFLAFVGGQPPALEKLKGKG	0.090	17	57	Yes
P01	C	26	LCMT2	chr15	43621964	Frameshift deletion	GQFMLQHFRQLNSPCMAWSVFLTWRRSGAA	0.070	47	60	Yes
P01	A	28	TNFRSF11A	chr18	60027241	SNV	VEHHGTEKSDVVCSSSLPARKPPNGN	0.070	6	25	Yes
P02	-	2	PSMD10	chrX	107330945	SNV	LKMIHILLYYKASTKIQDTEGNTPL	0.493	13	29	No
P02	B	3	KRAS	chr12	25380275	SNV	KKKLDILDTAGHEEYSAMRDQYMRTGE	0.420	2	11	Yes
P02	C	4	RNF220	chr1	45110737	SNV	GEAKEREALWGAVLNGGPPSTRITP	0.320	10	34	Yes
P02	C	5	TTLL11	chr9	124584976	SNV	LVCGRGVPSGGRPPHCGPPQEPSPS	0.303	7	9	Yes
P02	B	6	SMARCA4	chr19	11095980	SNV	HQMHKPMESMHEKGMLDDPRYNQMKGMGMRS	0.290	4	7	Yes
P02	A	7	RAP1GAP2	chr17	2911425	SNV	PEEDKFENGGHGGFVESFKRAIRVRSHSME	0.273	10	54	Yes
P02	-	8	TNS1	chr2	218712290	SNV	KEATSDPSWTPEEEPLNLEKKKKK	0.270	7	0	No
P02	C	9	MYO9A	chr15	72144565	SNV	TDAESVNLDDYNILVIASVFKQWLRDLPNKKK	0.268	12	48	Yes
P02	A	15	LSG1	chr3	194362826	SNV	GNRNKKEKSCRLYKHLDM	0.208	7	14	Yes
P02	B	16	PIK3CA	chr3	178936091	SNV	QLKAISTRDPLSEITKQEKDFLWSHRHYCVT	0.195	7	29	Yes
P02	A	17	POLR3GL	chr1	145460212	SNV	ASQGGGRGRGRGQLTFNV	0.193	2	8	Yes
P02	C	19	PTPRCAP	chr11	67203432	SNV	GEGDQQCGEASSPEQVPVRKKK	0.188	2	10	Yes
P02	-	20	SNX6	chr14	35044969	SNV	LKLSDLLKYYLRESQVAKDLLYRRSRSLVDY	0.175	17	58	No
P02	A	21	ARSI	chr5	149677313	SNV	ISEGRASPRTEILHNFDPLYNHAQHGSLEGG	0.175	16	36	Yes
P03	-	1	MIF	chr22	23894500	SNV	MPMFIVNTSVPRASVPDGFLS	0.388	16	19	No
P03	C	2	HADHB	chr2	26289946	SNV	KKCAAGGQGHAMIVEAYLK	0.303	8	8	Yes
P03	A	3	PQBP1	chrX	48902476	Frameshift deletion	GKERRHPPGGAGSLSQEQEGSKPK	0.295	14	30	Yes
P03	-	4	TP53	chr17	7675143	SNV	VDSTPPPGTRIRAMAIYKQSQHMTEV	0.213	15	34	No
P03	A	7	PSMD3	chr17	39996222	SNV	AKAIRDGVIEASINHKKGYVQSKEMID	0.158	12	42	Yes
P03	B	9	DNM2	chr19	10833261	SNV	YRAAGGEGSGLPPPIKEQRPKK	0.130	7	5	Yes
P03	B	10	SPAG16	chr2	213375036	Frameshift insertion	HSREKENDHQKVLLRKVFVKPGNKTNVKQR	0.110	16	31	Yes
P03	C	11	MED13L	chr12	116006330	SNV	TPVPDGKNAMSLFSSATKTDVRQDN	0.100	19	38	Yes
P03	-	13	ANO6	chr12	45439718	SNV	EYLALLPRLGHSGMISAHCNLRLPVD	0.083	21	44	No
P03	C	14	FCER1G	chr1	161220408	Frameshift insertion	EKSDGVYTMESPSITQAGVQWHDLGSLQKK	0.070	22	46	Yes
P03	A	16	GPSM1	chr9	136341025	Frameshift deletion	AQGEFQGCGGVLLHRHGPHQELRRGG	0.070	13	29	Yes
P03	B	17	GPSM1	chr9	136341025	Frameshift deletion	RHGPHQELRRGGIEARPAWRRWQPPENG	0.070	22	38	Yes
P03	C	18	GPSM1	chr9	136341025	Frameshift deletion	ARPAWRRWQPPENGAYKPVLLGLRDSTGL	0.070	38	24	Yes
P03	A	19	SNX21	chr20	45838289	SNV	KDPPSKYVRQGLAMLARLVSNSWP	0.070	12	36	Yes
P03	A	20	SGSM3	chr22	40404316	SNV	PLMEDAPQRLQWQAHLEFTHNHDVG	0.070	15	32	Yes
P04	C	1	MEN1	chr11	64572018	SNV	PEGGSTAQVPAPAASPPPEGPVLKK	0.335	8	3	Yes
P04	B	2	MYH14	chr19	50726572	SNV	TENTKKVIQYLAHVALSPKGRKEPGV	0.180	13	34	Yes
P04	-	3	TP53	chr17	7578479	SNV	PVQLWVDSTSPPGTRVRAMAIYKQS	0.165	12	6	No
P04	A	4	ANKRD11	chr16	89351239	SNV	KKAWSEVSSLSDSTRARLTSESDY	0.145	8	6	Yes
P04	A	5	RPL23	chr17	37004296	SNV	LRKKVLLCHPGWSAVTPLKLTVTLNKK	0.143	14	24	Yes
P04	B	6	KRAS	chr12	25398284	SNV	TEYKLVVVGADGVGKSALTIQLIQNH	0.130	7	6	Yes
P04	A	8	RBM25	chr14	73572607	Frameshift deletion	RERERTRARTRTGARERAR	0.100	7	0	Yes
P04	B	9	RBM25	chr14	73572607	Frameshift deletion	RAREGTGARKRKRQKTGPRRR	0.100	6	0	Yes
P04	-	10	ZEB1	chr10	31810505	SNV	SLPKQQGELLQRSTITSVYQNSVYSV	0.087	12	26	No
P04	A	11	C15orf40	chr15	83657573	Frameshift deletion	KSDVVLDKGLTFSPRLEHSGTTSGH	0.070	11	15	Yes
P04	B	12	C15orf40	chr15	83657573	Frameshift deletion	HSGTTSGHCSLDFPGSNNPPTSASQV	0.070	12	0	Yes
P04	A	13	C15orf40	chr15	83657573	Frameshift deletion	ASQVAGTTGACHPHPANFRIF	0.070	16	0	Yes
P04	B	15	PCTP	chr17	53864996	SNV	SNPLASASQSAGITGVSHHTRPIRAEPS	0.060	15	7	Yes
P04	C	17	PPP1CB	chr2	29004735	Frameshift deletion	FCCHGGRLVNLPYRFFFLLLFGKYLKKKK	0.060	26	34	Yes
P04	-	18	SMG5	chr1	156247808	Frameshift deletion	LRNKLRELCVKLMFCTQWTMGERLRS	0.050	17	21	No
P04	C	20B	SMG5	chr1	156247808	Frameshift deletion	SLSRLTKSTSTAGALWNVPTGRTWL	0.050	23	11	Yes
P05	-	31	RBM10	chrX	47041437	Frameshift deletion	RLPRTWNAGPAVSTNKKKTSKIASSLSAPC	0.200	18	56	No
P05	-	7	MYH9	chr22	36716404	SNV	FYYLLSGAGEHLKTELLLEPYNKYRFLSNG	0.173	12	48	No
P05	A	8	DISC1	chr1	231902938	SNV	GDDTHTPLRMEPRLLQPTAQDSLHVSITRRD	0.160	9	27	Yes
P05	-	9	PSTK	chr10	124740063	SNV	RKRGLCVPCGLPAAGKSTFARAL	0.147	9	18	No
P05	B	16	MFSD12	chr19	3567148	SNV	LGSSDSPASASRAVGITEPVPAGGGCR	0.130	5	37	Yes
P05	B	17	EDEM2	chr20	33703379	SNV	SGPWEPPARPRTLFSPENHDQ	0.127	7	0	Yes
P05	A	23	CPS1	chr2	211441079	SNV	KKGEVVFNTGLGGYPDAITDPAYK	0.113	4	20	Yes
P06	-	1	KRAS	chr12	25398284	SNV	TEYKLVVVGAVGVGKSALTIQLIQNHK	0.197	9	43	No
P06	A	2	NLRC5	chr16	57095846	SNV	SFPTLEACALGFKKKKKKERKKVSE	0.180	8	33	Yes
P06	-	3	PDZRN3	chr3	73450100	SNV	KKKKKKYIGDIHQDMDREELELEEVDLYR	0.143	10	5	No
P06	-	4	GALNS	chr16	88891240	SNV	DRPIFYYRGDTLMASTLGQHKAHFWTWTNS	0.140	11	53	No
P06	-	5	LILRB1	chr19	55146175	SNV	KKKLLLLLLLLFLLLRHRRQGKHWTSTQR	0.140	19	44	No
P06	C	6	LILRB1	chr19	55146175	SNV	LLFLLLRHRRQGKHWTSTQR	0.140	4	20	Yes
P06	B	7	SRSF6	chr20	42089202	SNV	RSRRRSRSRSRRSSHSRSRSISKSRSRSR	0.140	8	17	Yes
P06	B	9	NCAPH2	chr22	50956050	SNV	KKKKQLSSVQEDRANVVASSGVPQEAENEF	0.137	6	37	Yes
P06	A	10	STAG1	chr3	136068029	SNV	VRNKKGQPPLHKKR	0.137	7	0	Yes

Supplementary Table 6. The best response of each peptide/peptide pool for all 7 patients					
Patient ID	Experimental control / Number of peptide	Immunizing pools	IFN-γ spots	IFN-γ spots per 10^5 PBMCs	Response
P01	Positive control	/	1200	480	/
	Negative control	/	16	6	/
	2	C	484	194	Strong Positive
	3	A	598	239	Strong Positive
	5	C	441	176	Strong Positive
	6	B	702	281	Strong Positive
	7	C	543	217	Strong Positive
	9	A	588	235	Strong Positive
	10	B	347	139	Strong Positive
	12	B	406	162	Strong Positive
	15	A	337	135	Strong Positive
	16	B	245	98	Strong Positive
	26	C	176	59	Strong Positive
	28	A	113	38	Strong Positive
P02	Positive control	/	626	250	/
	Negative control	/	16	6	/
	3	B	172	69	medium Positive
	4	C	150	60	medium Positive
	5	C	180	72	medium Positive
	6	B	164	66	medium Positive
	7	A	156	62	medium Positive
	9	C	145	58	medium Positive
	15	A	150	60	medium Positive
	16	B	164	66	medium Positive

	17	A	60	30	weak Positive
	19	C	67	34	weak Positive
	21	A	40	20	weak Positive
P03	Positive control	/	1240	620	/
	Negative control	/	1	1	/
	2	C	2	1	negative
	3	A	3	2	negative
	7	A	4	2	negative
	9	B	4	2	negative
	10	B	5	3	negative
	11	C	3	2	negative
	14	C	2	1	negative
	16	A	1	1	negative
	17	B	108	54	medium Positive
	18	C	111	56	medium Positive
	19	A	101	51	medium Positive
	20	A	99	50	medium Positive
P04	Positive control	/	334	167	/
	Negative control	/	6	3	/
	1	C	18	6	negative
	2	B	31	10	weak Positive
	4	A	22	7	negative
	5	A	37	12	weak Positive
	6	B	28	9	negative
	8	A	23	8	negative
	9	B	26	9	negative
	11	A	10	3	negative
	12	B	12	4	negative
	13	A	38	13	weak Positive

	15	B	23	8	negative
	17	C	20	7	negative
	20B	C	23	8	negative
P05	Positive control	/	196	98	/
	Negative control	/	48	24	/
	8	A	82	41	negative
	16	B	91	46	negative
	17	B	137	69	negative
	23	A	158	79	negative
P06	Positive control	/	675	338	/
	Negative control	/	1	1	/
	2	A	7	3	negative
	6	C	5	2	negative
	7	B	4	2	negative
	9	B	1	0	negative
	10	A	0	0	negative
	12	A	0	0	negative
	13	A	0	0	negative
	15	B	0	0	negative
	16	B	2	1	negative
	17	C	6	2	negative
	18	C	5	2	negative
	19	A	12	5	negative
	20	C	6	2	negative
P07	Positive control	/	976	488	/
	Negative control	/	1	1	/
	22B	A	0	0	negative
	23	A	0	0	negative
	25	B	0	0	negative
	27	B	0	0	negative
	32	A	0	0	negative

Supplementary Table 7. Cytokine titers in peripheral blood for each patient

Fold change of cytokines in peripheral blood post vaccination									
Cytokines	Unit	P01	P02	P04	P07	P03	P05	P06	t-test
TNF-α	pg/ml	1.38	0.67	N/A	1.05	0.00	0.92	0.93	0.327
IL-1β	pg/ml	1.00	1.00	N/A	1.00	1.00	1.00	1.00	N/A
IL-6	pg/ml	1.38	1.32	N/A	0.75	1.10	14.66	1.55	0.359
IL-8	pg/ml	0.35	0.72	N/A	N/A	1.50	0.18	1.53	0.431
IL-10	pg/ml	1.00	1.00	N/A	2.47	1.00	1.00	1.00	0.374
IL-2	pg/ml	1.00	1.00	N/A	1.05	1.00	1.00	1.00	0.374
IFN-γ	pg/ml	4.53	9.20	N/A	4.64	1.36	1.00	0.81	0.031

Supplementary Table 8. T-cell subsets before and after vaccination

Fold Change post-vac

Marker	Unit	P01	P02	P04	P07	P03	P05	P06	t-test
CD4+ CD45RA+ CCR7+	Naïve CD4+T Cell (9.56-29.87%) (154.2-239.7 per ul)	1.06	1.25	1.21	0.92	0.92	1.20	0.45	0.259
CD4+ CD45RA- CCR7+	Central Memory CD4+T Cell (7.19-26.33%) (106.2-196.9 per ul)	0.81	0.84	0.93	1.13	1.11	0.92	0.10	0.461
CD4+ CD45RA+ CCR7-	Effector CD4+T Cell (0.44-6.06%) (6.432-23.10 per ul)	2.32	1.03	1.95	0.66	0.78	0.95	0.08	0.142
CD4+ CD45RA- CCR7-	Effector Memory CD4+T Cell (17.27-39.12%) (149.4-242.1 per ul)	2.36	1.96	1.52	0.63	0.70	0.85	0.22	0.081
CD4+ CD38+ HLA-DR+	Activated CD4+T Cell (0.58-2.43%) (8.757-14.33 per ul)	0.77	0.79	1.70	0.73	1.34	0.56	0.25	0.507
CD8+ CD45RA+ CCR7+	Naïve CD8+T Cell (1.23-8.84%) (22.80-74.10 per ul)	1.16	0.91	0.77	0.42	0.59	1.95	0.57	0.628
CD8+ CD45RA- CCR7+	Central Memory CD8+T Cell (0.37-1.63%) (7.909-12.64 per ul)	2.26	0.46	0.44	1.50	3.68	5.32	0.24	0.217
CD8+ CD45RA+ CCR7-	Effector CD8+T Cell (3.26-10.42%) (28.02-52.05 per ul)	0.80	1.47	1.66	0.64	0.57	1.37	0.63	0.469
CD8+ CD45RA- CCR7-	Effector Memory CD8+T Cell (8.08-27.96%) (50.34-98.14 per ul)	1.24	1.88	1.31	0.64	0.54	0.72	1.01	0.174
CD8+ CD38+ HLA-DR+	Activated CD8+T Cell (0.27-5.70%) (8.462-16.54 per ul)	0.72	1.25	2.59	1.16	0.69	1.92	1.79	0.951

Baseline

Marker	Unit	P01	P02	P04	P07	P03	P05	P06	t-test
CD4+ CD45RA+ CCR7+	Naïve CD4+T Cell (9.56-29.87%) (154.2-239.7 per ul)	87.80	90.23	286.09	42.32	233.06	20.06	22.20	0.706
CD4+ CD45RA- CCR7+	Central Memory CD4+T Cell (7.19-26.33%) (106.2-196.9 per ul)	268.94	165.37	106.73	260.51	91.58	53.58	46.08	0.035
CD4+ CD45RA+ CCR7-	Effector CD4+T Cell (0.44-6.06%) (6.432-23.10 per ul)	6.62	129.65	7.26	39.53	8.07	4.81	2.56	0.290
CD4+ CD45RA- CCR7-	Effector Memory CD4+T Cell (17.27-39.12%) (149.4-242.1 per ul)	193.53	191.70	133.97	266.09	165.69	82.06	68.58	0.077
CD4+ CD38+ HLA-DR+	Activated CD4+T Cell (0.58-2.43%) (8.757-14.33 per ul)	28.62	49.35	8.43	80.08	4.84	6.40	16.34	0.137
CD8+ CD45RA+ CCR7+	Naïve CD8+T Cell (1.23-8.84%) (22.80-74.10 per ul)	14.31	11.25	77.16	9.63	21.43	14.44	8.98	0.533
CD8+ CD45RA- CCR7+	Central Memory CD8+T Cell (0.37-1.63%) (7.909-12.64 per ul)	2.14	8.34	5.58	5.83	1.17	0.99	1.75	0.041
CD8+ CD45RA+ CCR7-	Effector CD8+T Cell (3.26-10.42%) (28.02-52.05 per ul)	291.15	65.22	70.16	412.31	197.25	69.05	39.78	0.369
CD8+ CD45RA- CCR7-	Effector Memory CD8+T Cell (8.08-27.96%) (50.34-98.14 per ul)	201.86	68.93	106.99	350.47	61.35	25.99	28.68	0.113
CD8+ CD38+ HLA-DR+	Activated CD8+T Cell (0.27-5.70%) (8.462-16.54 per ul)	55.97	19.71	13.75	130.00	8.81	4.12	6.86	0.188

Day 22

Marker	Unit	P01	P02	P04	P07	P03	P05	P06	t-test
CD4+ CD45RA+ CCR7+	Naïve CD4+T Cell (9.56-29.87%) (154.2-239.7 per ul)	93.45	112.97	345.29	39.05	213.43	23.99	9.97	0.532
CD4+ CD45RA- CCR7+	Central Memory CD4+T Cell (7.19-26.33%) (106.2-196.9 per ul)	218.26	139.02	99.07	293.26	101.42	49.40	4.48	0.060
CD4+ CD45RA+ CCR7-	Effector CD4+T Cell (0.44-6.06%) (6.432-23.10 per ul)	15.36	132.92	14.18	26.22	6.29	4.57	0.21	0.257
CD4+ CD45RA- CCR7-	Effector Memory CD4+T Cell (17.27-39.12%) (149.4-242.1 per ul)	457.14	374.85	203.38	166.62	116.72	69.88	15.17	0.041
CD4+ CD38+ HLA-DR+	Activated CD4+T Cell (0.58-2.43%) (8.757-14.33 per ul)	21.94	39.09	14.33	58.39	6.50	3.60	4.14	0.057
CD8+ CD45RA+ CCR7+	Naïve CD8+T Cell (1.23-8.84%) (22.80-74.10 per ul)	16.67	10.23	59.47	4.09	12.68	28.13	5.09	0.665
CD8+ CD45RA- CCR7+	Central Memory CD8+T Cell (0.37-1.63%) (7.909-12.64 per ul)	4.83	3.79	2.47	8.74	4.30	5.27	0.42	0.458
CD8+ CD45RA+ CCR7-	Effector CD8+T Cell (3.26-10.42%) (28.02-52.05 per ul)	232.74	95.65	116.48	264.07	112.63	94.49	25.02	0.125
CD8+ CD45RA- CCR7-	Effector Memory CD8+T Cell (8.08-27.96%) (50.34-98.14 per ul)	249.63	129.79	140.21	224.64	33.00	18.72	29.05	0.007
CD8+ CD38+ HLA-DR+	Activated CD8+T Cell (0.27-5.70%) (8.462-16.54 per ul)	40.36	24.74	35.59	150.26	6.08	7.91	12.30	0.181

Supplementary Table 9. Mutation of KRAS for each patient

Patient ID	KRAS mutation	Chr	Position	Ref_bp	Alt_bp	AA_Change	Mutation_type	Included in iNeo-Vac-P01
P01	No	-	-	-	-		-	-
P02	Yes	chr12	25380275	T	A	Q61H	SNV	Yes
P03	Yes	chr12	25245350	C	T	G12D	SNV	Yes
P04	Yes	chr12	25398284	C	T	G12D	SNV	Yes
P05	No	-	-	-	-		-	-
P06	Yes	chr12	25398284	C	A	G12V	SNV	Yes
P07	Yes	chr12	25245350	C	A	G12V	SNV	Yes
